# Distinct synaptic properties of perisomatic inhibitory cell types and their different modulation by cholinergic receptor activation in the CA3 region of the mouse hippocampus

**DOI:** 10.1111/j.1460-9568.2010.07292.x

**Published:** 2010-06

**Authors:** Gergely G Szabó, Noémi Holderith, Attila I Gulyás, Tamás F Freund, Norbert Hájos

**Affiliations:** 1 Laboratory of Network Neurophysiology, Institute of Experimental Medicine, Hungarian Academy of SciencesH-1083 Budapest, Hungary; 2 Laboratory of Cerebral Cortex Research, Institute of Experimental Medicine, Hungarian Academy of SciencesBudapest, Hungary

**Keywords:** GABA, inhibition, interneuron, IPSC, synchronization

## Abstract

Perisomatic inhibition originates from three types of GABAergic interneurons in cortical structures, including parvalbumin-containing fast-spiking basket cells (FSBCs) and axo-axonic cells (AACs), as well as cholecystokinin-expressing regular-spiking basket cells (RSBCs). These interneurons may have significant impact in various cognitive processes, and are subjects of cholinergic modulation. However, it is largely unknown how cholinergic receptor activation modulates the function of perisomatic inhibitory cells. Therefore, we performed paired recordings from anatomically identified perisomatic interneurons and pyramidal cells in the CA3 region of the mouse hippocampus. We determined the basic properties of unitary inhibitory postsynaptic currents (uIPSCs) and found that they differed among cell types, e.g. GABA released from axon endings of AACs evoked uIPSCs with the largest amplitude and with the longest decay measured at room temperature. RSBCs could also release GABA asynchronously, the magnitude of the release increasing with the discharge frequency of the presynaptic interneuron. Cholinergic receptor activation by carbachol significantly decreased the uIPSC amplitude in all three types of cell pairs, but to different extents. M2-type muscarinic receptors were responsible for the reduction in uIPSC amplitudes in FSBC– and AAC–pyramidal cell pairs, while an antagonist of CB_1_ cannabinoid receptors recovered the suppression in RSBC–pyramidal cell pairs. In addition, carbachol suppressed or even eliminated the short-term depression of uIPSCs in FSBC– and AAC–pyramidal cell pairs in a frequency-dependent manner. These findings suggest that not only are the basic synaptic properties of perisomatic inhibitory cells distinct, but acetylcholine can differentially control the impact of perisomatic inhibition from different sources.

## Introduction

Although in cortical structures only every fifth neuron is GABAergic (Somogyi *et al.*, 1998), these neurons significantly influence information processing in neuronal networks (Miles *et al.*, 1996; Pouille & Scanziani, 2004). GABAergic cells express distinct sets of proteins and give rise to characteristic dendritic and axonal arbours, leading to functional diversity (Freund & Buzsaki, 1996; Klausberger & Somogyi, 2008). Based on target preference, cortical GABAergic cells with local axonal projections can be divided into two major categories: cells innervating predominantly either the perisomatic membranes or the dendrites of principal neurons (Buhl *et al.*, 1994; Miles *et al.*, 1996); this study does not address the latter category. The perisomatic region is defined as the domain of the plasma membrane which includes the proximal dendrites, the cell body and the axon initial segment (AIS; Freund & Buzsaki, 1996). This region is targeted by three types of inhibitory neurons in cortical areas, namely by the parvalbumin (PV)-expressing fast-spiking basket cells (FSBCs) and axo-axonic cells (AACs) as well as by the cholecystokinin (CCK)-containing regular-spiking basket cells (RSBCs). The basket cells innervate the somata and proximal dendrites (Blackstad & Flood, 1963) whereas the AACs target the AISs of pyramidal neurons (Somogyi, 1977).

Perisomatic inhibitory cells can effectively control the generation of sodium-dependent action potentials, and thereby determine the output of principal cells (Cobb *et al.*, 1995; Miles *et al.*, 1996; Szabadics *et al.*, 2006). As different types of perisomatic inhibitory cells have been found to be distinctly recruited during local network operation (Glickfeld & Scanziani, 2006), and their behaviour is also dissimilar during various oscillatory activities (Klausberger *et al.*, 2003, 2005), these GABAergic cells probably accomplish distinct functions in information processing.

Acetylcholine can substantially regulate the function of perisomatic inhibitory cells by affecting their membrane properties or by modulating their GABA release (Lawrence, 2008), and thus altering network dynamics (Hasselmo, 2006). While CCK- but not PV-containing interneurons in the neocortex can be depolarized by cholinergic receptor activation (Kawaguchi & Kubota, 1997), GABA release from both cell types is depressed by cholinergic receptor agonists, although by different mechanisms (Fukudome *et al.*, 2004). AACs have not been distinguished from basket cells in these studies so it is unknown how their GABA release is affected by acetylcholine.

As the three perisomatic inhibitory cell types have similar dendritic and axonal arborizations, they cannot be unequivocally identified at the light-microscopic level. Usually PV-containing interneurons are separated from CCK-expressing GABAergic cells based on their neurochemically different characters and/or their physiological properties (Kawaguchi & Kubota, 1997; Pawelzik *et al.*, 2002; Hefft & Jonas, 2005), but the distinction between FSBCs and AACs was only possible by analyzing their targets using electron microscopy (Gulyas *et al.*, 1993). In this study, we identified AACs at the light-microscopic level by double-staining to visualize their targets, i.e. the AISs. Using whole-cell recordings, we examined the basic properties of unitary inhibitory postsynaptic currents (uIPSCs) in perisomatic inhibitory interneuron–pyramidal cell pairs and the short-term plasticity of these connections. In addition, we investigated the effect of cholinergic receptor activation on the properties and dynamics of uIPSCs.

## Materials and methods

### Experimental animals and slice preparation

All experiments were carried out in accordance with the Hungarian Act of Animal Care and Experimentation (1998, XXVIII, section 243/1998), and with the guidelines of the institutional ethical code. Transgenic mice expressing enhanced green fluorescent protein (eGFP) controlled by glutamate decarboxylase 65 (GAD65) promoter (Lopez-Benedito *et al.*, 2004) or PV promoter (Meyer *et al.*, 2002) were used. Mice (postnatal days 15–23) were deeply anaesthetized with isoflurane and decapitated. The brain was quickly removed and placed into ice-cold cutting solution containing (in mm): sucrose, 252; KCl, 2.5; NaH_2_PO_4_, 1.25; MgCl_2_, 5; CaCl_2_, 0.5; NaHCO_3_, 26; and glucose, 10. The cutting solution was bubbled with 95% O_2_ and 5% CO_2_ (carbogen gas) for at least half an hour before use. Horizontal hippocampal slices (200–300 μm thick) were prepared using a Leica VT 1000S or a VT1200S microtome (Leica, Nussloch, Germany), and kept in an interface-type holding chamber at room temperature for at least 60 min before recording in standard ACSF with the composition (in mm) NaCl, 126; KCl, 2.5; NaH_2_PO_4_, 1.25; MgCl_2_, 2; CaCl_2_, 2; NaHCO_3_, 26; and glucose, 10. Solutions were prepared with ultra pure water and bubbled with carbogen gas.

### Paired recordings

**Fig. 7:**
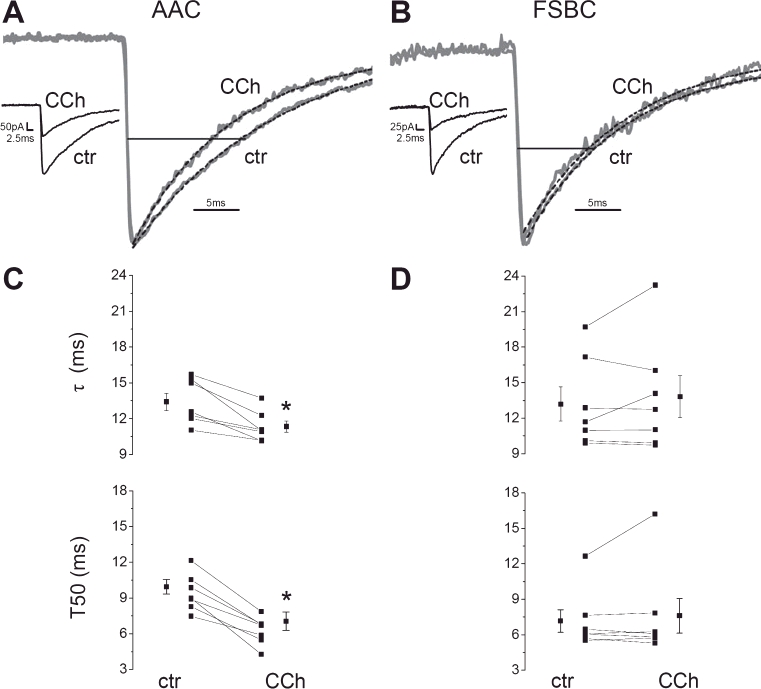
Synaptic cross-talk between release sites of AACs, but not those of FSBCs, may explain the differences in decay kinetics. (A) Two representative superimposed IPSCs originating from an AAC–pyramidal cell pair in control conditions (ctr) and in the presence of carbachol (CCh). Dashed lines represent the exponential fits while the horizontal line illustrates the decay width at 50% (T50) of the peak amplitude. Note that the traces are normalized in order to visualize the changes in the decay. Original traces are shown in the inserts. (B) Same as in A, but with uIPSCs originating from an FSBC cell. (C) Summary of changes in decay time constants (τ) and T50 values at AAC–pyramidal cell pairs in response to carbachol. (D) Same as in C, but for FSBC–pyramidal cell pairs. Each data point represents a cell pair in the line series. Data calculated from seven paired recordings in each case; *P < 0.01.

Slices were transferred to a submersion type of recording chamber. To reduce the occurrence of spontaneous synaptic events, the flow rate was 2–3 mL/min. Experiments were performed at room temperature under visual guidance using an Olympus microscope (BX61WI; Olympus Corp., Tokyo, Japan). Fluorescence of eGFP-containing cells was excited by a monochromator at 488 nm wavelength or by standard epifluorescence using a UV lamp, and the resulting fluorescence visualized with a CCD camera (TILL photonics, Gräfelfing, Germany, or Hamamatsu Photonics, Japan). Whole-cell patch-clamp recordings were made using a Multiclamp 700B amplifier (Molecular Devices), filtered at 2 kHz, digitized at 5 kHz with a PCI-6024E board (National Instruments, Austin, TX, USA), recorded with in-house data acquisition and stimulus software (Stimulog, courtesy of Professor Zoltán Nusser, Institute of Experimental Medicine, Hungarian Academy of Sciences) and analyzed off-line using the evan software (courtesy of Professor István Mody, UCLA, CA). Patch pipettes were pulled from borosilicate glass tubing with resistances of 3–6 MΩ. The intracellular solution used for the presynaptic cell contained (in mm) K-gluconate, 110; NaCl, 4; Mg-ATP, 2; HEPES, 40; and GTP, 0.3; with 0.2% biocytin; adjusted to pH 7.3 using KOH and with an osmolarity of 290 mOsm/L. The intrapipette solution used for the postsynaptic cell contained (in mm) CsCl, 80; Cs-gluconate, 60; MgCl_2_, 1; Mg-ATP, 2; NaCl, 3; HEPES, 10; and QX-314 [2(triethylamino)- *N*-(2,6-dimethylphenyl) acetamine], 5; adjusted to pH 7.3 with CsOH, and with an osmolarity of 295 mOsm/L. Presynaptic interneurons were held in current-clamp mode around a membrane potential of −65 mV, and stimulated by brief current pulses (1.5 ms, 1–2 nA). Pyramidal cells were clamped at a holding potential of −65 mV. Series resistance was frequently monitored and compensated between 65–75%, and cells that changed > 25% during recording were discarded from further analysis. For the analysis of the kinetic properties of uIPSCs we used only those recordings where series resistance changed by ≤ 10% (Fig. 7, Table 2). In experiments with carbachol, 5 μm NBQX was occasionally added to the bath solution to reduce the high background synaptic activity.

**Table 2:** Summary of uIPSC properties before and after carbachol treatment

	FSBC				AAC			
	Control	Carbachol	*P*-value	*n*	Control	Carbachol	*P*-value	*n*
Peak amplitude (pA)	367.4 ± 167.5	155.2 ± 78.7*	0.008	8	509.8 ± 103.7	116.5 ± 26.5*	0.004	9
Rise time (10–90%, ms)	1.2 ± 0.2	1.9 ± 0.6*	0.04	8	0.9 ± 0.1	1.2 ± 0.3	0.07	9
T50 (ms)	7.1 ± 1.0	7.6 ± 1.5	0.94	7	9.5 ± 0.6	6.3 ± 0.4*	0.02	7
Decay τ (ms)	13.2 ± 1.4	13.8 ± 1.8	0.74	7	13.4 ± 0.7	11.3 ± 0.5*	0.008	7
Probability of failure	0.089 ± 0.049	0.194 ± 0.069	0.06	8	0.027 ± 0.022	0.258 ± 0.064*	0.008	9
Latency (ms)	1.5 ± 0.1	1.4 ± 0.1	0.15	8	1.6 ± 0.2	1.8 ± 0.2	0.09	9

* Statistical significance obtained by Wilcoxon signed-rank paired test. AAC, axo-axonic cell; FSBC, fast-spiking basket cell; uIPSC, unitary IPSC.

### *Post hoc* anatomical identification of interneurons

After recording, slices were fixed in 4% paraformaldehyde in 0.1 m phosphate buffer (PB; pH 7.4) for at least 60 min, followed by washout with PB several times, cryoprotected in 20% sucrose and repeatedly freeze–thawed (for details see Gulyas *et al.*, 1993). Biocytin was visualized using avidin-biotinylated horseradish peroxidase complex reaction (ABC; Vector Laboratories, Burlingame, CA, USA) with nickel-intensified 3,3-diaminobenzidine as a chromogen. After dehydration and embedding in Durcupan (Fluka), neurons were identified based on their dendritic and axonal arborization and some representative cells were reconstructed with the aid of a drawing tube using a 40× objective.

### Identification of FSBCs and AACs using double immunofluorescent labelling

After recordings, the slices were fixed as above, washed, cryoprotected, embedded in agar (1%) and re-sectioned at 60 μm thickness. Every third section was processed for electron microscopy where biocytin was visualized as above. The sections were then treated in 1% OsO_4_, followed by 1% uranyl acetate, dehydrated in a graded series of ethanol, and embedded in epoxy resin (Durcupan; Fluka). Ultrathin sections of 60 nm thickness were cut for electron microscopy, and the postsynaptic targets of 5–10 boutons of each examined cells were identified. The remaining sections were processed for fluorescent double immunolabelling. They were treated with 0.2 mg/mL pepsin (Cat. No.: S3002; Dako) in 0.2 m HCl at 37°C for 5 min and were washed in 0.1 m PB similar to the procedure developed by Watanabe *et al.* (1998). Sections were blocked in normal goat serum (NGS; 10%) made up in Tris-buffered saline (TBS, pH = 7.4) followed by incubations in mouse anti-Ankyrin-G (1 : 100; Santa Cruz Biotechnology) diluted in TBS containing 2% NGS and 0.3% Triton X-100. Following several washes in TBS, Cy3-conjugated goat antimouse (1 : 500; Jackson) was used to visualize the immunoreaction, while Alexa488-conjugated streptavidin (1 : 500; Invitrogen) to visualize the biocytin. Sections were then mounted on slides in Vectashield (Vector Laboratories). Images were taken using an AxioImager Z1 axioscope (Carl Zeiss MicroImaging GmbH, Germany).

### Data analysis and materials

The kinetic properties of uIPSCs were investigated on averaged events that were calculated with excluding the transmission failures. The latency of synaptic transmission was calculated by subtracting the time of the action potential peaks from the start of the postsynaptic currents. This latter value was estimated by subtracting the rise time from the peak time of events calculated from the time of the action potential peaks. Calculation of asynchronous release was achieved by the comparison of the average charge (area under the curve) of all currents in a 100-ms-long time window before and after the action potential trains. Fitting of single exponential functions on the decaying phases of averaged uIPSCs and statistical analyses were performed using Origin 8.0 software (OriginLab Corporation, Northampton, MA, USA). As most data in this work did not have a Gaussian distribution according to the Shapiro–Wilk’s *W* test or the Kolmogorov–Smirnov test, nonparametric statistics were used. Multiple groups of data were compared using the nonparametric Kruskal–Wallis anova test completed with comparison of samples as pairs with the Mann–Whitney *U*-test. For dependent samples the Wilcoxon signed-ranks test was used. *P <*0.05 was considered a significant difference. Data are presented as mean ± SEM.

All chemicals and drugs were purchased from Sigma Aldrich (St Louis, MO, USA), except AM251 and AF/DX 116, which were obtained from Tocris (Bristol, UK).

## Results

### Identification of different types of perisomatic interneurons

To investigate the synaptic properties of the three types of perisomatic inhibitory neurons in the CA3 region of the hippocampus, it was indispensable to unequivocally separate them from each other. RSBCs were sampled in *in vitro* slices prepared from transgenic mice in which eGFP expression was under the control of the GAD65 promoter. As in this mouse line PV-containing interneurons do not express eGFP at all in the hippocampus (Lopez-Benedito *et al.*, 2004), we used another transgenic mouse to specifically target the FSBCs and AACs. In this mouse line the bacterial artificial chromosome technique was used to drive the expression of eGFP selectively in PV-containing cells (Meyer *et al.*, 2002), providing a tool to obtain recordings from hippocampal FSBCs and AACs (Katsumaru *et al.*, 1988).

**Fig. 1:**
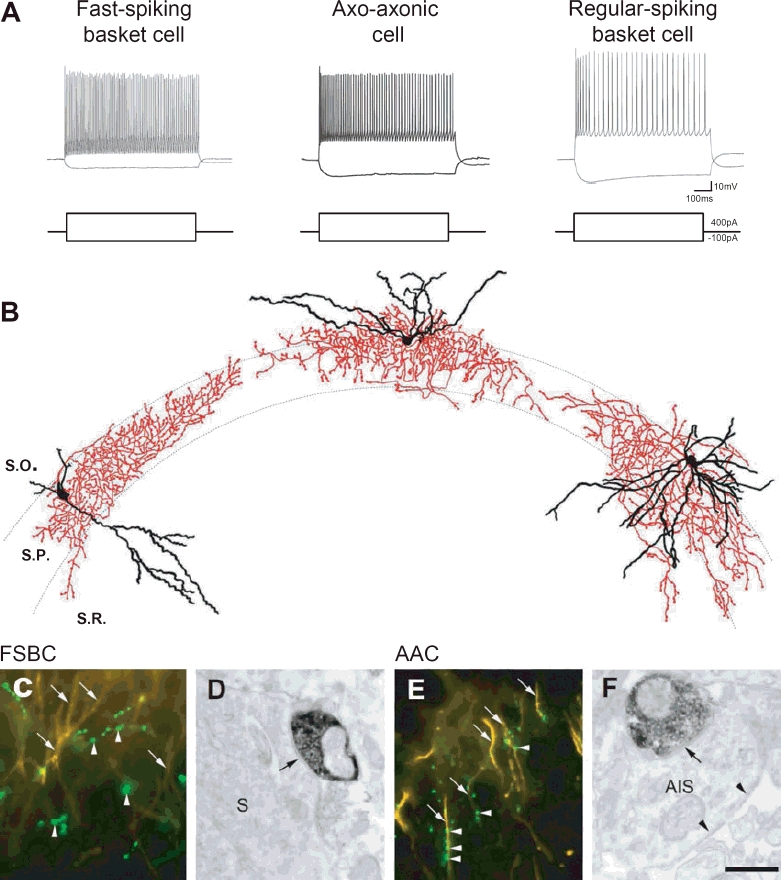
Electrophysiological and morphological properties of the three types of perisomatic inhibitory interneurons in the CA3 region of the mouse hippocampus. (A) Firing characteristics of three representative cells in response to depolarizing and hyperpolarizing current steps of 400 pA and −100 pA, respectively. (B) Camera lucida reconstructions of representative biocytin-loaded neurons. Left, fast-spiking basket cell (FSBC); middle, axo-axonic cell (AAC); right, regular-spiking basket cell (RSBC). Cell bodies and dendrites of interneurons are shown in black and axon clouds in red. (C) Double immunofluorescent labelling for biocytin (green; arrowheads point to labelled terminals) and for ankyrin-G (yellow; arrowheads point to labelled AISs) shows no close appositions of biocytin-filled boutons with AISs. (D) An electron micrograph of a peroxidase-labelled axon terminal of the same cell as in C illustrates that this bouton formed symmetrical synapses (arrow) on CA3 pyramidal cell soma(s), a characteristic of basket cells. (E) Double immunofluorescent labelling as in C shows tight appositions of biocytin-labelled boutons (green; arrowheads) and ankyrin-G-labelled AISs (yellow; arrows), suggesting that this neuron is an AAC. (F) An electron micrograph of a peroxidase-labelled axon ending of the same cell as in E confirms that this interneuron formed synapses (arrow) on AISs of CA3 pyramidal cells, indicating that the recorded neuron was an AAC. Arrowheads show undercoating, characteristic of AISs. Scale bar in F, 100 μm (B), 15 μm (C and E) and 0.5 μm (D and F).

All the recorded interneurons were tested for firing characteristics and filled with biocytin to allow *post hoc* visualization of their morphology (Fig. 1A and B). Only those neurons identified in this study as RSBCs sampled from GAD65-eGFP mice, which had an axonal arbour predominantly in the stratum pyramidale surrounding somata, and had typical regular firing, were used (*n*=17; Daw *et al.*, 2009). To distinguish FSBCs and AACs after recordings obtained in slices from PV-eGFP mice, double immunofluorescent staining was performed to visualize the biocytin-filled axon collaterals together with the AISs of neurons, which were labelled with an antibody developed against ankyrin-G. This scaffolding protein is present in high concentrations in the AIS of neurons, where it anchors several proteins, including voltage-gated sodium channels (Nav1.2 and 1.6; Jenkins & Bennett, 2001), so it is appropriate to visualize AISs at the light microscopic level (Boiko *et al.*, 2007). We observed two clearly distinguishable patterns of labelling in the double-stained materials. There were cells with biocytin-filled axons that only rarely approached ankyrin-G-stained profiles (*n*=23, Fig. 1C), whereas other cells had axon collaterals forming close appositions with ankyrin-G-labelled segments, often in a climbing fiber-like manner (*n*=26, Fig. 1E). To confirm that intracellularly-labelled boutons avoiding ankyrin-G-immunoreactive elements derived from basket cells, as suggested by the morphology, electron microscopic examination was performed. In all cases tested, we found that axon terminals of these neurons formed symmetrical synapses on the somata or proximal dendrites of CA3 pyramidal cells (*n*=5; Fig. 1D), therefore these interneurons were confirmed to be basket cells. In those cases in which biocytin-filled boutons surrounded ankyrin-G-immunopositive segments, electron microscopic studies confirmed that axon terminals formed synaptic contacts on the AIS of pyramidal cells (*n*=5; Fig. 1F); as a result, we identified these interneurons as AACs. In PV-eGFP mice, we also recorded four bistratified cells and one oriens–lacunosum moleculare cell, which was not unexpected as previous data indicated that these GABAergic cell types could express PV at low levels (Klausberger & Somogyi, 2008). These neurons were excluded from this study.

Hence, using anatomical methods, we identified all the three types of perisomatic inhibitory interneurons whose synaptic outputs have been investigated.

### Basic synaptic properties of connections between perisomatic inhibitory cells and postsynaptic pyramidal neurons

**Fig. 2:**
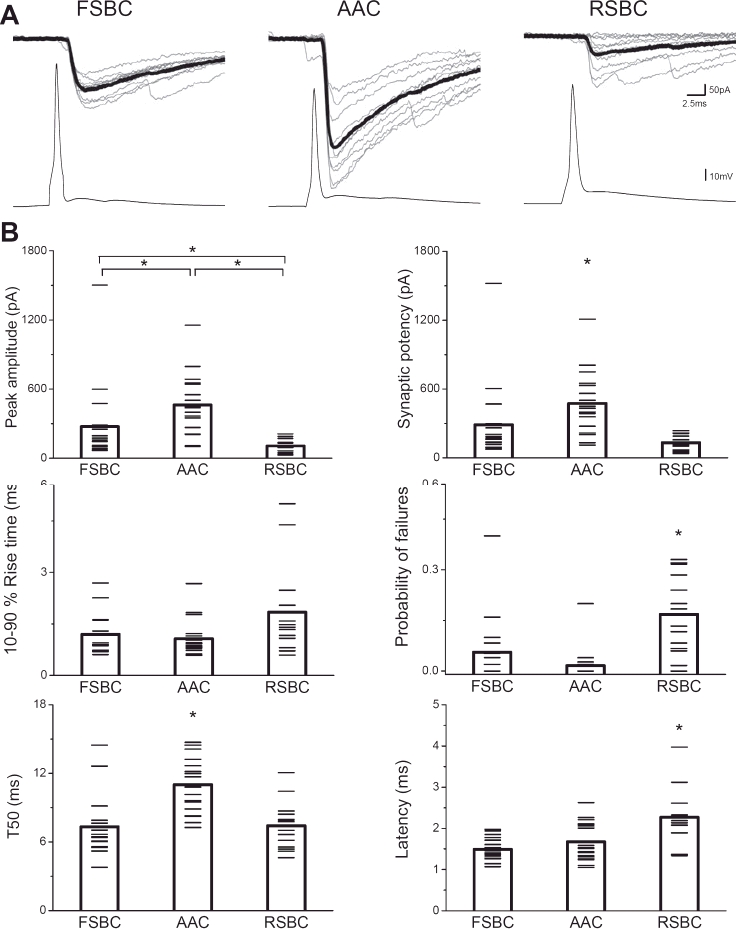
Basic properties of synaptic communication between perisomatic inhibitory interneurons and pyramidal cells. (A) Ten superimposed uIPSCs (thin lines) evoked by single presynaptic action potentials in representative FSBC–, AAC– and RSBC–pyramidal cell pairs. Averages of uIPSCs are indicated by thick black lines. (B) Comparison of the peak amplitude, the synaptic potency, the 10–90% rise time, the failure probability, the half-decay (T50) and the latency obtained in the three types of perisomatic inhibitory interneurons and pyramidal cell pairs. Bars show the averages and short lines show the mean values for individual cell pairs. Asterisks indicate significant differences (see Table 1).

In the first set of experiments we investigated the basic properties of synapses formed by the three types of perisomatic inhibitory cells on their pyramidal cell targets. To this end, uIPSCs were recorded from synaptically coupled perisomatic inhibitory cell–pyramidal neuron pairs in the CA3 region. We compared the peak amplitude (including failures), potency (excluding failures), 10–90% rise time and T50 values (i.e. the width of currents at the half of the peak amplitude) of uIPSCs. In addition, the synaptic latency of transmission (i.e. the time between the action potential peak and the beginning of the postsynaptic current) and the probability of transmission failure were also calculated (Fig. 2; Table 1). The analysis showed that the three groups are different regarding peak amplitude (*H*_2,50_ = 22.36; *P*<0.0001), synaptic potency (*H*_2,50_ = 18.8; *P*<0.0001), probability of failures (*H*_2,50_ = 20.83; *P*<0.0001), T50 value (*H*_2,48_ = 18.2; *P*=0.0001) and latency (*H*_2,49_ = 14.23; *P*=0.0008) whereas 10–90% rise time values belonging to the three groups did not differ significantly from each other (*H*_2,49_ = 4.15; *P*=0.12). Further statistical investigations revealed that in AAC–pyramidal cell pairs synaptic currents had the largest peak amplitude and potency, as did T50 values of postsynaptic currents; these parameters significantly differed from the values of the other two groups (Fig. 2; Table 1). Moreover, synaptic transmission of RSBCs was found to have a higher probability of failures and longer latency than either FSBCs or AACs (Fig. 2; Table 1). These results indicate that the synaptic output of the individual AACs could have the largest potential to influence the activity of pyramidal neurons in the CA3 region of the hippocampus.

**Table 1:** Summary of uIPSC properties

	FSBC			AAC			RSBC		
Parameter	Data	*P*-value*	*n*	Data	*P*-value†	*n*	Data	*P*-value‡	*n*
Peak amplitude (pA)	274.9 ± 84.7	0.004	17	463.3 ± 61.8	< 0.0001	18	107.1 ± 13.9	0.049	15
Potency (pA)	288.4 ± 84.3	0.006	17	475.5 ± 63.8	< 0.0001	18	133.0 ± 16.2	0.08	15
Rise time (10–90%, ms)	1.2 ± 0.1	0.36	17	1.1 ± 0.1	0.05	18	1.8 ± 0.3	0.23	14
T50 (ms)	7.3 ± 0.6	0.0002	17	11.0 ± 0.6	0.0005	17	7.4 ± 0.5	0.74	14
Probability of failure	0.056 ± 0.025	0.06	17	0.016 ± 0.011	< 0.0001	18	0.168 ± 0.029	0.002	15
Latency (ms)	1.5 ± 0.1	0.31	17	1.7 ± 0.1	0.008	18	2.3 ± 0.2	0.0002	14

*P*-values represent the results of the statistical comparison of

* FSBC vs. AAC

† AAC vs. RSBC and

‡ RSBC vs. FSBC using the Mann–Whitney *U*-test. AAC, axo-axonic cell; FSBC, fast-spiking basket cell; RSBC, regular-spiking basket cell; uIPSC, unitary IPSC.

### Carbachol, an ACh receptor agonist, reduced the amplitudes of uIPSCs to different extents depending on the presynaptic cell type

**Fig. 3:**
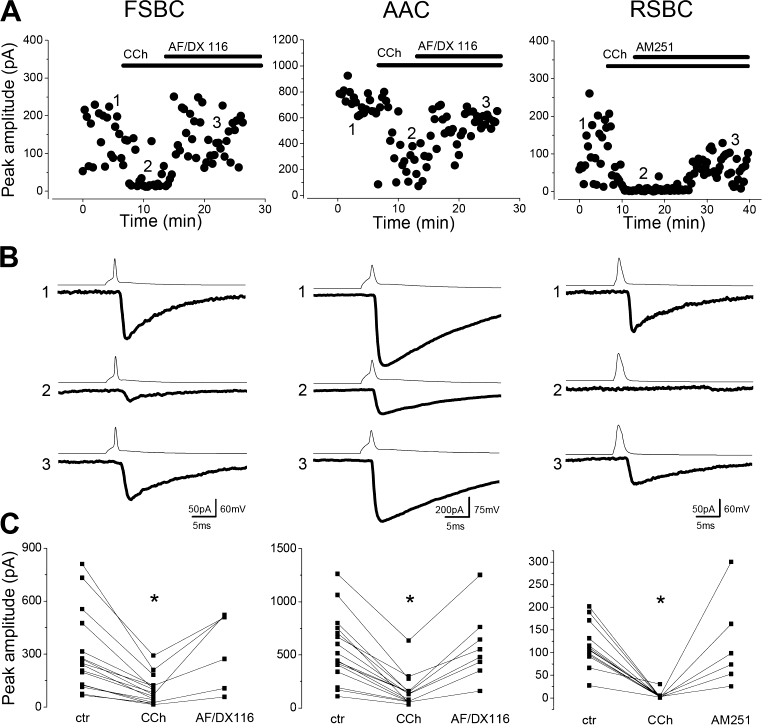
Cell-type-specific suppression of uIPSC amplitudes by the ACh receptor agonist carbachol. (A) Representative experiments obtained in FSBC–, AAC– and RSBC–pyramidal cell pairs. In each case, bath application of 5 μm carbachol suppressed the uIPSC amplitude. In FSBC– and AAC–pyramidal cell pairs the reduction in the peak amplitude could be restored by an M2-receptor-preferring antagonist AF/DX 116 (10 μm), while a CB_1_ cannabinoid receptor antagonist AM251 (1 μm) reversed the suppression of uIPSC amplitude in RSBC–pyramidal cell pairs. (B) Average of five uIPSCs evoked by single presynaptic action potentials taken at the labelled time points. (C) Summary data of all pairs. Each square represents a mean value of IPSC amplitude from individual cell pairs under a given condition. Asterisks indicate the significant differences. Note the different scales on *y* axis.

Hippocampal circuits are extensively supplied by cholinergic fibers arriving from the medial septum (Wenk *et al.*, 1975; Wainer *et al.*, 1985). To investigate the effect of cholinergic receptor activation on the perisomatic inhibition, we obtained paired recordings during pharmacological activation of ACh receptors by bath application of carbachol (2–5 μm). First we examined the postsynaptic effect of carbachol in the different types of perisomatic inhibitory cells. The analysis revealed that cholinergic receptor activation similarly changed the membrane potential of all types of perisomatic inhibitory cells (*H*_2,26_ = 4.81; *P*=0.09). Carbachol depolarized FSBCs by 6.1 ± 1.3 mV (*n*=8), AACs by 3.6 ± 2.0 mV (*n*=11) and RSBCs by 6.3 ± 1.1 mV (*n*=7). Next, we investigated the presynaptic effect of carbachol by looking into the properties of uIPSCs. In these sets of experiments transmission failures were included in the average uIPSCs. Carbachol caused a robust decrease in uIPSC amplitudes in all three types of cell pairs. In FSBC–pyramidal cell pairs and in AAC–pyramidal cell pairs, the synaptic currents were reduced to 29.9 ± 2.5% (*n*=16, *P*<0.0001, Fig. 3) and 27.1 ± 2.8% of control amplitude (*n*=16, *P*<0.0001, Fig. 3), respectively. In contrast, carbachol caused an almost total suppression of neurotransmission in RSBC–pyramidal cell pairs, reducing the amplitude to 6.0 ± 3.4% of control (*n*=13, *P*<0.0001). Accordingly, the magnitude of the reduction in the amplitude caused by carbachol proved to be dissimilar among different types of cell pairs (*H*_2,45_ = 19.98; *P*<0.0001). Whereas the magnitude of the suppression in FSBC– and AAC–pyramidal cell pairs was similar (*P*=0.44), both differed significantly from the results obtained in RSBC–pyramidal cell pairs (*P*=0.0001). Next, we investigated the nature of receptors involved in the reduction in uIPSCs. In slices prepared from PV-eGFP mice, a muscarinic receptor antagonist AF/DX 116, which prefers M2-type receptors, was tested. In FSBC–pyramidal cell pairs, CCh decreased the amplitudes of uIPSCs to 28.3 ± 3.6% of control (*n*=6, *P*=0.03), an effect that was restored by the antagonist to 95.6 ± 19.7% of control (*n*=6, *P*=0.31). Similarly, in AAC–pyramidal cell pairs the amplitudes were reduced to 30.2 ± 4.0% of control by CCh (*n*=8, *P*=0.008), a decrease that could be reversed with AF/DX 116–101.6 ± 12.4% of control (*n*=8, *P*=0.38). As shown earlier, carbachol may trigger the synthesis of endocannabinoids via M1/M3 muscarinic receptors; these endocannabinoid signalling molecules could reduce GABA release from RSBC terminals by activating CB_1_ cannabinoid receptors (Fukudome *et al.*, 2004; Neu *et al.*, 2007). Therefore, we tested whether the suppression of release at these synapses can be reversed by antagonizing CB_1_ receptor function. In RSBC–pyramidal cell pairs, the amplitude of uIPSCs was reduced to 2.9 ± 0.7% of control (*n*=6, *P*=0.03); this was completely reversed by the co-application of a CB_1_ receptor antagonist AM251 (104.6 ± 39.2% of control, *n*=6, *P*=0.84; Fig. 3).

These data demonstrate that carbachol effectively decreases the GABA release from perisomatic inhibitory cells to a different extent via different mechanisms, suggesting that the network dynamics could be substantially affected by cholinergic septal input and also via controlling the contribution of distinct cell types to perisomatic inhibition.

### Carbachol reduces the short-term depression at FSBC– and AAC–pyramidal cell synapses in a frequency-dependent manner

**Fig. 4:**
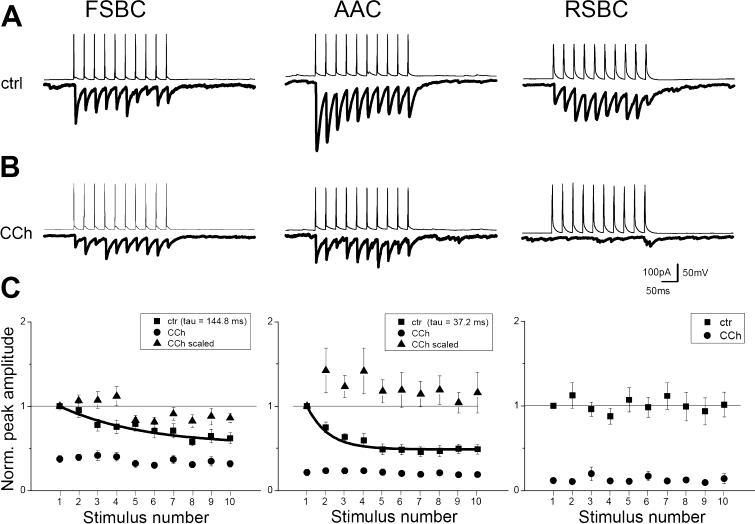
Carbachol changed the short-term dynamics of transmitter release. (A) Representative averaged IPSCs in response to 10 action potentials at a frequency of 30 Hz in control conditions. (B) Responses in the same pairs in the presence of 5 μm carbachol. (C) Summary of changes in release dynamics effected by carbachol at 30 Hz recorded in FSBC– (*n* = 14), AAC– (*n* = 17) and RSBC–pyramidal cell pairs (*n* = 14). Filled squares represent normalized peak amplitudes in control conditions, circles show the same in carbachol, and triangles show data obtained in carbachol that were normalized to the first IPSC amplitude in carbachol. Amplitudes are plotted against time during trains. Curves represent exponential fit to control data points. Note that RSBC data were unsuitable for fitting with exponentials because of the lack of short-term plasticity.

**Fig. 5:**
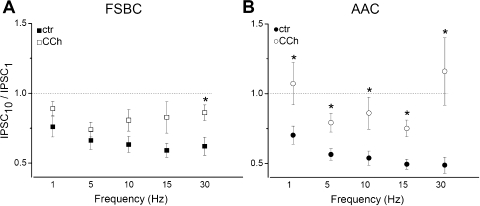
Frequency-dependent changes in short-term plasticity of synaptic transmission in FSBC– and AAC–pyramidal cell pairs. (A) Ratio of IPSC_10_/IPSC_1_ is shown at different frequency values at FSBC–pyramidal cell pairs. (B) Same as in A, but for AAC–pyramidal cell pairs. Solid squares and circles represent control conditions, open symbols show data from carbachol-treated slices. All data are from 14 FSBC– and 17 AAC–pyramidal cell pairs; *P < 0.05.

In the next set of experiments we studied the short-term changes of uIPSC amplitudes evoked by action potential trains with distinct frequencies and sought to determine the effect of carbachol on the dynamics of synapses. To this end, uIPSCs were recorded in response to 10 action potentials evoked at frequencies of 1, 5, 10, 15 and 30 Hz. An example of these experiments is presented in Fig. 4, where the discharges of presynaptic interneurons were elicited at 30 Hz. Synaptic currents in FSBC– and AAC–pyramidal cell pairs showed powerful depression during the trains (Fig. 4A), whereas the uIPSC amplitudes in this RSBC–pyramidal cell pair was facilitating and depressing (Fig. 4A). In contrast to FSBC– and AAC–pyramidal cell pairs, in which the typical depression of uIPSC amplitudes increased with the firing frequency of interneurons (Fig. 5), we observed that the dynamics of RSBC–pyramidal cell synapses were very heterogeneous regarding the short-term plasticity. These synapses showed depression, facilitation or facilitation–depression, which gave on average no change in the uIPSC amplitude at all tested frequencies (an example is shown for 30 Hz in Fig. 4C).

After performing the recordings in control conditions, carbachol was bath-applied. As expected in RSBC–pyramidal cell pairs, GABA release suffered almost full block upon cholinergic receptor activation, thus no further investigations of short-term changes of IPSCs could be performed. In the case of PV-containing interneuron–pyramidal cell pairs, the magnitude of the depression notably decreased as a result of carbachol treatment (Fig. 4B). We found that carbachol altered the synaptic depression in a frequency-dependent manner. In the case of FSBC–pyramidal cell pairs the extent of the depression decreased significantly at 30 Hz (*P*=0.01; *n*=14) and tended to decrease at the other tested frequencies (*P*=0.05 at 15 Hz, *P*=0.21 at 10 Hz, *P*=0.58 at 5 Hz and *P*=0.05 at 1 Hz), whereas at AAC–pyramidal cell connections the synaptic depression was reduced or even eliminated at all tested frequencies (*P*=0.0003 at 30 Hz, *P*=0.0002 at 15 Hz, *P*=0.017 at 10 Hz, *P*=0.015 at 5 Hz and *P*=0.001 at 1 Hz; *n*=17; Fig. 5).

These data suggest that cholinergic receptor activation not only changes the magnitude of perisomatic inhibition originated from FSBCs and AACs but also effectively regulates its short-term plasticity in a frequency-dependent manner.

### Asynchronous GABA release from RSBC terminals showed frequency dependence

**Fig. 6:**
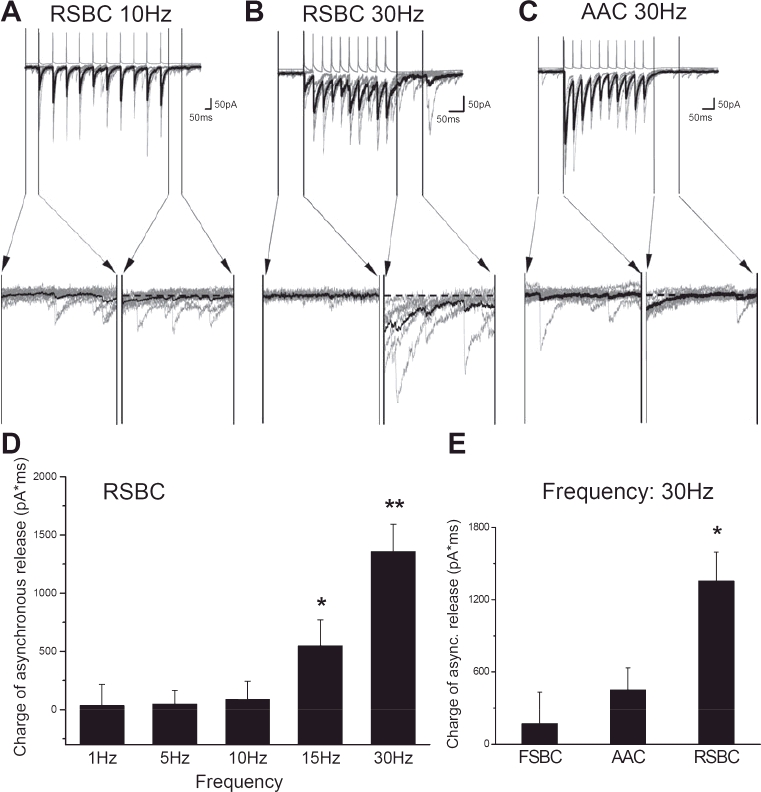
Asynchronous transmitter release in perisomatic inhibitory interneuron–pyramidal cell pairs. Examples of IPSC trains in response to 10 action potentials evoked at (A) 10 Hz and at (B) 30 Hz in an RSBC–pyramidal cell pair, and (C) at 30 Hz in an AAC–pyramidal cell pair. Averages are shown in black and the individual traces in grey. The magnified 100-ms-long periods before and after the action potential trains were compared to estimate the amount of asynchronous release, demonstrating the presence of asynchronous release in the RSBC–pyramidal cell pair only at 30 Hz. (D) Summary of the frequency-dependent asynchronous release in RSBC–pyramidal cell pairs. A significant asynchronous release was found at 15 and 30 Hz (*P < 0.05 and **P < 0.001, respectively). (E) Summary of asynchronous release in the three types of perisomatic inhibitory interneuron–pyramidal cell pairs at the discharge frequency of 30 Hz.

Previous studies reported that CCK-containing basket cells in the dentate gyrus or in the CA1 hippocampal region were capable of asynchronous transmitter release and, thus, could generate fluctuating and long-lasting inhibitory signals (Hefft & Jonas, 2005; Daw *et al.*, 2009). We also noticed in our experiments that the occurrence of IPSCs often increased after the action potential trains in RSBC–pyramidal cell pairs. Therefore, we investigated the magnitude of the asynchronous release as a function of the discharge frequency of the presynaptic RSBCs, and contrasted this with data obtained in FSBC– and AAC–pyramidal cell pairs. We compared the total charge transfer of spontaneous postsynaptic currents received by the pyramidal cells before and after the action potential trains elicited at different frequencies (Fig. 6). At RSBC–pyramidal cell synapses we observed robust asynchronous release that showed strong frequency dependence. While below 10 Hz no asynchronous release could be observed, at 15 Hz (*P*=0.02) and at 30 Hz (*P*=0.0001, *n*=14) significant increases in the charge transfer could be detected (Fig. 6). We then examined the possibility of asynchronous release at the other two types of perisomatic inhibitory cell, but we did not find any significant change in the charge transfer following action potential trains tested at 30 Hz (*P*=0.17 for FSBCs, *n*=17; and *P*=0.05 for AACs, *n*=18), confirming that neither FSBCs nor AACs release transmitter in an asynchronous manner. In addition, we tested whether the asynchronous release from RSBC terminals was also sensitive to carbachol. At 30 Hz the amount of asynchronous release drastically decreased in the presence of carbachol (22.2 ± 34.1% of control charge; *P*=0.001; *n*=14).

These results indicate that, in contrast to FSBCs and AACs, RSBCs can release GABA asynchronously; the magnitude of this release increases with the firing frequency of the interneurons and this type of release is also suppressed by cholinergic receptor activation.

### Synaptic cross-talk between terminals of AACs may elongate the decay of synaptic currents

We observed different decay kinetics of postsynaptic currents originating from AACs than from basket cells (Fig. 2). As we obtained our experiments at room temperature, when the neurotransmitter uptake is reduced (Binda *et al.*, 2002), we wondered whether the slower decay of synaptic currents recorded in AAC–pyramidal cell pairs might be due to the spillover of GABA between release sites, as they are in a close proximity along the AIS of pyramidal cells (Fig. 1E), providing the structural basis for synaptic cross-talk. One way to test this assumption is to compare the decay of IPSCs evoked under conditions with high release probabilities with those that were recorded under reduced release probabilities. In the latter case, GABA has a lower chance of reaching its receptors in the neighbouring synapses so the decay of IPSCs should be faster (Overstreet & Westbrook, 2003). Therefore, we determined the decay time constants (τ) by fitting an exponential to the averaged IPSCs in control conditions (i.e. with high release probability) and in the presence of carbachol (i.e. with lower release probability), as carbachol affects GABA release from the terminals without directly altering GABA receptor function (Behrends & ten Bruggencate, 1993). We found that the decay of uIPSCs was significantly faster at AAC–pyramidal cell synapses in the presence of carbachol than in control conditions (*P*=0.01, *n*=7, Fig. 7A and C; Table 2). Similar results were obtained by analyzing the half-width (T50) values of uIPSCs in control conditions and in the presence of carbachol (*P*=0.01; *n*=7, Fig. 7A and C; Table 2). In contrast, both types of analysis failed to detect any difference between the decay of uIPSCs recorded in FSBC–pyramidal cell pairs under conditions with high and low release probabilities (*P*=1.0 and *P*=0.93 for the comparison of τ and T50 values, respectively; *n*=7; Fig. 7B and D; Table 2). The comparison of the uIPSC decays of AACs with that of FSBCs in the presence of carbachol revealed no difference (*P*=0.46 for τ and T values, *n*=7 for FSBC and AACs), suggesting that kinetics of GABA receptor operation might be similar at the two types of synapses if they are operating independently of each other.

These data are in line with the hypothesis that, when GABA uptake is compromised, synaptic cross-talk could significantly elongate the synaptic inhibition originating from AACs but not from FSBCs.

## Discussion

In this study we showed that AACs can be unequivocally distinguished from basket cells at the light-microscopic level using double immunofluorescent staining to visualize biocytin-filled axon collaterals and AISs with ankyrin-G. Paired recordings obtained from perisomatic inhibitory cells and pyramidal neurons revealed that AACs produced uIPSCs with the largest amplitudes and with the longest decays, probably due to the spillover of GABA between release sites under our recording conditions. In contrast to AACs and FSBCs, RSBCs produced uIPSCs that had the longest latency, the probability of transmitter release was the lowest at these connections, and they could release GABA asynchronously, the magnitude of release increasing with the discharge frequency of RSBCs. Cholinergic receptor activation by carbachol reduced the amplitude of uIPSCs that were recorded in FSBC– and AAC–pyramidal cell pairs, an effect that could be fully restored by the M2-type muscarinic receptor-preferring antagonist. In contrast, carbachol muted the synaptic transmission in RSBC–pyramidal cell pairs via triggering endocannabinoid production, as antagonism of CB_1_ cannabinoid receptors reversed the reduction in IPSC amplitude. In addition, the depressing nature of synaptic currents in FSBC– and AAC–pyramidal cell pairs were largely reduced, or even eliminated, by carbachol in a frequency-dependent manner.

As GABA_A_ receptors present at the perisomatic inhibitory synapses consist of similar subunits (alpha 1, alpha 2, beta 2 and gamma 2; Nusser *et al.*, 1996; Nyiri *et al.*, 2001; Kasugai *et al.*, 2006), neither the larger amplitude nor the longer decay of uIPSCs in AAC–pyramidal cell pairs compared to those recorded in basket cell–pyramidal cell pairs could be the result of distinct subunit compositions. In addition to the similar subunit composition, the area of synapses formed by basket cells and AACs was found to be similar (Nusser *et al.*, 1998). Thus, the large amplitude might reflect the large number of synaptic contacts in these cell pairs. Although the number of axon terminals of AACs contacting CA3 pyramidal cells has not been studied specifically in CA3, basket cells preferentially innervate pyramidal cells in CA3 via two to six synapses (Miles *et al.*, 1996; Biro *et al.*, 2006). This number is lower than the observations obtained in other regions, for instance in CA1, where 10–12 synaptic contacts were identified between basket cells and pyramidal cells (Buhl *et al.*, 1994; Cobb *et al.*, 1997). In this region, AACs have been shown to innervate their targets via similar numbers of synapses (Maccaferri *et al.*, 2000), which is in agreement with physiological measurements showing that basket cells and AACs in CA1 produce uIPSCs with similar amplitude. If we assume that the sum of GABA_A_ receptor conductances in a given synapse that originate from perisomatic inhibitory cells are similar in different regions, then in CA3 more synaptic contacts should be responsible for the larger IPSC amplitude in AAC–pyramidal cell pairs than in basket cell–pyramidal cell connections.

By reducing the GABA release from the axon terminals of both AACs and FSBCs, carbachol accelerated the decay of uIPSCs recorded only in AAC– but not in FSBC–pyramidal cell pairs. This finding agrees well with previous data showing that GABA molecules could spill over to neighbouring release sites in the case of AAC– but not in FSBC–pyramidal cell pairs when the transmitter uptake is blocked by uptake inhibitors (Overstreet & Westbrook, 2003). As we performed the experiments at room temperature, i.e. when uptake systems were largely altered (Binda *et al.*, 2002), the significantly longer decay time of uIPSCs of AACs observed in the present study could also be due to the cross-talk of neighbouring synapses. Under physiological conditions (i.e. at higher temperature), GABAergic synapses of AACs are not subject to cross-talk because produce IPSCs with similar kinetics to basket cells (Maccaferri *et al.*, 2000; Overstreet & Westbrook, 2003). Nevertheless the present observations might gain significance in pathological states in which the GABA uptake system is compromised (Volk *et al.*, 2001; Liu *et al.*, 2007).

We found that the synaptic depression at PV-containing interneuron–pyramidal cell connections observed under control conditions was largely reduced or even eliminated in the presence of carbachol. These results seemingly contradict those findings obtained in the dentate gyrus, where carbachol suppressed but did not abolish the depression (Hefft *et al.*, 2002). The major difference that could explain the disagreement between these two studies was the recording temperature. While we performed our experiments at room temperature, the study in the dentate gyrus was done at 34°C. As the vast majority of physiological processes, including the affinity of muscarinic acetlycholine receptors to their agonists (Aronstam & Narayanan, 1988), have been found to be altered by lowering the temperature, the higher affinity of carbachol with their receptors, causing more stable receptor–G-protein complex at room temperature, might produce a larger reduction in the release probability in parallel with the elimination of the depression (Brenowitz *et al.*, 1998). In agreement with this hypothesis, we observed a 70% reduction in the peak amplitude after carbachol application at room temperature, whereas at 34°C this agonist suppressed the first IPSC amplitude by only 30% (Hefft *et al.*, 2002). This temperature-dependent difference in the efficacy of carbachol in decreasing the initial release probability could account for its distinct effects on synaptic depression. Similarly, the slower decay of uIPSCs in our study compared to those values found at 33–34°C (Bartos *et al.*, 2002) is probably due to the difference in the recording temperature (Otis & Mody, 1992).

What could be the reason for the longer latency, lower release probability and asynchronous nature of GABA release characteristic of axon terminals originating from RSBCs than from PV-containing interneurons? Previous studies showed that Ca^2+^enters the terminals of FSBCs via P/Q-type voltage-gated Ca^2+^channels that are in the active zone of presynaptic terminals, where vesicles filled with transmitter molecules are located (Wilson *et al.*, 2001; Hefft & Jonas, 2005; Bucurenciu *et al.*, 2008). This mechanism allows the axon endings of FSBCs (and probably of AACs as well) to release GABA upon action potential discharge with high probability and with precise timing. In contrast, the axon terminals of RSBCs are equipped with N-type voltage-gated Ca^2+^channels that are probably located at a distance from active zone (Wilson *et al.*, 2001; Hefft & Jonas, 2005). The distant location of Ca^2+^entry from the release site could cause larger jitter and longer delay in the time of transmitter release, lower chance of releasing vesicles, and a build-up of Ca^2+^levels in the terminals that prolongs the transmitter release upon firing at high frequencies (present study; Rozov *et al.*, 2001; Wilson *et al.*, 2001; Hefft *et al.*, 2002; Hefft & Jonas, 2005; Daw *et al.*, 2009). These contrasting features in Ca^2+^entry into the boutons of PV-containing interneurons vs. RSBCs might, at least in part, explain the observed differences in IPSC characteristics, which predict their functional differences in neuronal operation (Freund & Katona, 2007).

In addition to the Ca^2+^entry, the signalling cascade triggered by cholinergic receptor activation that reduces GABA release from the axon terminals of PV-containing interneurons and RSBCs is also different. In the former case, the activation of M2-type muscarinic receptors located at the axon endings of FSBCs and AACs control GABA release (present study; Hajos *et al.*, 1998; Fukudome *et al.*, 2004). In contrast, carbachol has been shown to act on the postsynaptically located M1/M3 types of muscarinic receptors in pyramidal cells that triggers the synthesis of endocannabinoids, which act in a retrograde fashion via CB_1_ cannabionoid receptors located on the terminals of RSBCs, resulting in reduced GABA release (present study; Fukudome *et al.*, 2004; Neu *et al.*, 2007). In summary, the different efficacies of carbachol in the suppression of synaptic inhibition generated by the three types of perisomatic GABAergic cells could be due to the distinct signalling machinery underlying the reduction in vesicular release.

Our observation that carbachol could massively reduce or even eliminate the depression of IPSC amplitude in a frequency-dependent manner suggests that cholinergic tone originating from the medial septum may change the dynamics of information processing in the neuronal networks by altering the efficacy of feed-forward inhibition (Pouille & Scanziani, 2004; Pouille *et al.*, 2009). The mechanism underlying the change in short-term dynamics of synapses is probably related to the reduction in the initial release probability, an adjustment that promotes information transfer at higher frequencies (Brenowitz *et al.*, 1998).

### Functional implications

Although the three types of GABAergic cells investigated in this study innervate the perisomatic region of pyramidal cells, their function might be very different in neuronal information processing. This assumption is strongly supported by *in vivo* experiments which revealed that FSBCs, RSBCs and AACs discharged dissimilarly during different types of network oscillations in the CA1 region (Klausberger *et al.*, 2003, 2005), providing a temporal frame for dividing the labour among these inteneurons. The input and output properties of FSBCs are designed to respond and follow faithfully the ongoing network events (Hefft & Jonas, 2005; Doischer *et al.*, 2008; Hu *et al.*, 2010). For instance, by participating in both feed-forward and feed-back inhibition, they could set the dynamic range of cortical circuits or maintain oscillatory activities at different frequencies (Sik *et al.*, 1995; Freund & Katona, 2007; Pouille *et al.*, 2009). In contrast, RSBCs predominantly integrate the inputs from distinct sources and provide a prolonged, less precise GABAergic output that could modulate learning processes at cellular levels at different timescales (Carlson *et al.*, 2002; Hefft & Jonas, 2005; Glickfeld & Scanziani, 2006; Puighermanal *et al.*, 2009). As AACs specifically target AISs of principal cells, their role might be to regulate action potential generation directly. The fact that AACs are excitatory in cortex in certain states or inhibitory in others (Szabadics *et al.*, 2006), as in the hippocampus (Glickfeld *et al.*, 2009), does not influence the conclusion that they can play a role in rhythmic or intermittent synchronization of large pyramidal cell populations. As our data indicate, cholinergic input could affect the outputs of these GABAergic cells differently, so it is likely that this subcortical impact might also alter the network dynamics by controlling the inhibitory clamp provided by the functionally distinct types of perisomatic inhibitory cells.

## Abbreviations

AAC: axo-axonic cell
AIS: axon initial segment
CCK: cholecystokinin
eGFP: enhanced green fluorescent protein
FSBC: fast-spiking basket cell
GAD65: glutamate decarboxylase 65
IPSC: inhibitory postsynaptic current
PV: parvalbumin
RSBC: regular-spiking basket cell
uIPSC: unitary IPSC
